# Novel machine learning models to predict pneumonia events in supratentorial intracerebral hemorrhage populations: An analysis of the *Risa-MIS-ICH* study

**DOI:** 10.3389/fneur.2022.955271

**Published:** 2022-08-25

**Authors:** Yan Zheng, Yuan-Xiang Lin, Qiu He, Ling-Yun Zhuo, Wei Huang, Zhu-Yu Gao, Ren-Long Chen, Ming-Pei Zhao, Ze-Feng Xie, Ke Ma, Wen-Hua Fang, Deng-Liang Wang, Jian-Cai Chen, De-Zhi Kang, Fu-Xin Lin

**Affiliations:** ^1^Department of Neurosurgery, Neurosurgery Research Institute, The First Affiliated Hospital, Fujian Medical University, Fuzhou, China; ^2^Department of Neurosurgery, Binhai Branch of National Regional Medical Center, The First Affiliated Hospital, Fujian Medical University, Fuzhou, China; ^3^Fujian Institute for Brain Disorders and Brain Science, The First Affiliated Hospital, Fujian Medical University, Fuzhou, China; ^4^Fujian Provincial Clinical Research Center for Neurological Diseases, The First Affiliated Hospital, Fujian Medical University, Fuzhou, China; ^5^Department of Neurosurgery, Anxi County Hospital, Quanzhou, China; ^6^Clinical Research and Translation Center, The First Affiliated Hospital, Fujian Medical University, Fuzhou, China

**Keywords:** pneumonia, predict, machine learning, ensemble model, intracerebral hemorrhage, stroke

## Abstract

**Background:**

Stroke-associated pneumonia (SAP) contributes to high mortality rates in spontaneous intracerebral hemorrhage (sICH) populations. Accurate prediction and early intervention of SAP are associated with prognosis. None of the previously developed predictive scoring systems are widely accepted. We aimed to derive and validate novel supervised machine learning (ML) models to predict SAP events in supratentorial sICH populations.

**Methods:**

The data of eligible supratentorial sICH individuals were extracted from the *Risa-MIS-ICH* database and split into training, internal validation, and external validation datasets. The primary outcome was SAP during hospitalization. Univariate and multivariate analyses were used for variable filtering, and logistic regression (LR), Gaussian naïve Bayes (GNB), random forest (RF), K-nearest neighbor (KNN), support vector machine (SVM), extreme gradient boosting (XGB), and ensemble soft voting model (ESVM) were adopted for ML model derivations. The accuracy, sensitivity, specificity, and area under the curve (AUC) were adopted to evaluate the predictive value of each model with internal/cross-/external validations.

**Results:**

A total of 468 individuals with sICH were included in this work. Six independent variables [nasogastric feeding, airway support, unconscious onset, surgery for external ventricular drainage (EVD), larger sICH volume, and intensive care unit (ICU) stay] for SAP were identified and selected for ML prediction model derivations and validations. The internal and cross-validations revealed the superior and robust performance of the GNB model with the highest AUC value (0.861, 95% CI: 0.793–0.930), while the LR model had the highest AUC value (0.867, 95% CI: 0.812–0.923) in external validation. The ESVM method combining the other six methods had moderate but robust abilities in both cross-validation and external validation and achieved an AUC of 0.843 (95% CI: 0.784–0.902) in external validation.

**Conclusion:**

The ML models could effectively predict SAP in sICH populations, and our novel ensemble model demonstrated reliable robust performance outcomes despite the populational and algorithmic differences. This attempt indicated that ML application may benefit in the early identification of SAP.

## Introduction

Stroke-associated pneumonia (SAP) is the most common infectious complication in spontaneous intracerebral hemorrhage (sICH) individuals, with an estimated incidence of 15–25% in overall stroke populations (1–3). SAP is usually adversely associated with increased mortality, prolonged hospital stays, and poor prognosis (3–5). The current large phase III clinical trials have not found the benefits of routine antibiotic prevention for general stroke individuals (6, 7). Therefore, the accurate prediction and early intervention of SAP might contribute to improving the prognosis. Thus, a reliable model is needed for predicting and monitoring potential SAP, so that exact prophylactic interventions or therapeutic antibiotics can be tailored promptly.

In recent decades, a few studies have indicated several independent risk factors for SAP, including older age (5, 8–13), male sex (8, 9, 13, 14), severe stroke (4, 5, 8–16), intubation (4, 15), nasogastric feeding or dysphagia (4, 8, 16), and deeper location and larger volume of sICH (4, 11, 15). Some of these variables were included in several predictive scoring systems for SAP risk stratifications, such as the A^2^DS^2^ and PNA scores in Germany (9, 12), and the AIS/ICH-APS scores in China (10, 11), and the ISAN score in the UK (13). However, most scoring systems are designed for acute ischemic stroke (AIS) populations (9, 10, 12, 13), and none of the SAP prediction scoring systems are widely accepted in routine clinical practice.

At present, prediction models based on machine learning (ML) have been applied to predict the occurrence and prognosis of various diseases, which greatly promoted diagnostic performance and facilitated more responsive health systems (17–19). In clinical applications, ML algorithms are applied for risk stratification and prognosis prediction of disease and guide clinicians to apply corresponding measures timely. Compared to traditional scoring systems, ML models show smarter, more accurate, more timely, and more convenient characteristics (18–21). While there is currently no ML model for SAP forecasting. Thus, we aim to derive and validate novel supervised ML models to predict SAP events in supratentorial sICH populations and expect to develop a superior and automatic tool for clinical practice.

## Materials and methods

### Study design and participants

The data for this analysis were obtained from the retrospective database of the *Risk Stratification and Minimally Invasive Surgery in Acute Intracerebral Hemorrhage Patients* (*Risa-MIS-ICH*) study (Clinical Trials Identifier: NCT03862729, https://www.clinicaltrials.gov), which was a multicenter ambispective cohort study. Two centers were involved in the retrospective cohort for this work, including the First Affiliated Hospital, Fujian Medical University (FAHFMU, Fuzhou, Fujian), and Anxi County Hospital (ACH, Quanzhou, Fujian). The FAHFMU subcohort (from January 2015 to July 2020) was for the variable filtrations and model derivations/validations. The ACH subcohort (from June 2019 to April 2021) was introduced into this work for external validation. The study protocol followed the principles of the Declaration of Helsinki and was approved by the ethics committee of FAHFMU (GN: MRCTA, ECFAH of FUM [2018]082) and documented in each center. No informed consent was required for the retrospective cohort. This work was reported in accordance with the Transparent Reporting of a Multivariable Prediction Model for Individual Prognosis or Diagnosis (TRIPOD) statement (22).

The inclusion and exclusion criteria of the participants are shown as follows:

*Inclusion criteria:*

- Diagnosed with spontaneous sICH by computed tomography (CT)/CT angiography (CTA) scan, and the interval time from onset to recorded CT/CTA scan ≤ 48 h;
- Glasgow Coma Scale (GCS) score > 5 and no cerebral herniation at admission;
- Onset age ≥ 18 years.

*Exclusion criteria:*

- With any intracranial etiology of supratentorial hemorrhage of arteriovenous malformation (AVM), arterial aneurysm, hemorrhagic cerebral tumor stroke, hemorrhagic infarction, coagulation disorders, or any other potential organic lesions indicating nonspontaneous sICH;
- Occurrence of infratentorial hemorrhage;
- Evidence of pregnancy, or pre-stroke life expectancy < 3 months.

*Additional criteria for SAP prediction model derivations/validations in this work:*

- Interval time from onset to admission ≤ 24 h;
- Hospital stay ≥ 48 h;
- Receiving no mechanical ventilation or ventilation time ≤ 24 h before SAP events;
- Underwent recent pulmonary infectious disease or received any antibiotic therapy ≤ 4 weeks;
- Critical data loss about SAP in the laboratory, imaging, or other important clinical information.

According to the present guideline, the diagnosis of sICH participants required radiologic records and exclusion of other organic lesions causing hemorrhage. Only supratentorial sICH participants were enrolled in the *Risa-MIS-ICH* study, and the participants with the cerebral herniation or low GCS scores usually indicated poor prognosis, which was excluded from the study scope. The exclusion of the juvenile and the pregnant population is for ethical consideration. Furthermore, for the unbiased diagnosis of SAP and the precise analysis, the strict additional criteria had to exclude short-term hospitalization, infection associated with mechanical ventilation, and undefined participants.

**Figure 1:**
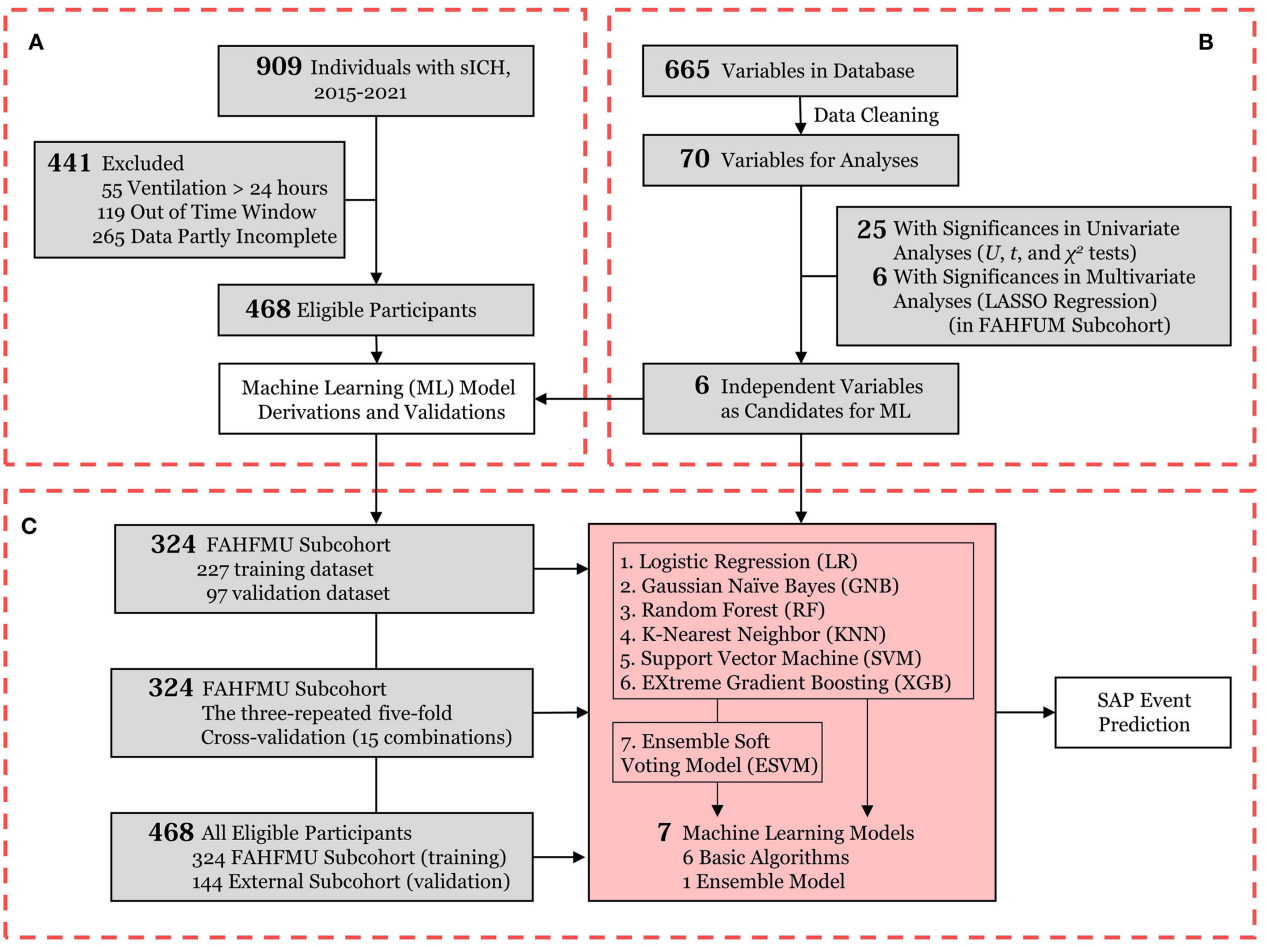
Flowchart of the current work. **(A)** Participant enrollment in the retrospective cohort of the *Risa-MIS-ICH* study; **(B)** Data flow from the FAHFMU subcohort; **(C)** The prediction model derivations and internal/cross-/external validations for SAP events. sICH, supratentorial intracerebral hemorrhage; ML, machine learning; LASSO, least absolute shrinkage and selection operator; SAP, stroke-associated pneumonia.

The screening process of this work is presented in Figure 1A.

### Variable extractions and primary outcomes

Relevant information about participants were retrieved from the electronic medical record (EMR) systems from each neurological research center. The electronic data capture (EDC, http://61.154.9.209:8090/, RealData Corporation, Ningbo, Zhejiang, China) system was employed for database establishment and data collection. The trained professional clinical research coordinators (CRCs) were commissioned for data entry and follow-up. The *Risa-MIS-ICH* database included 665 variables and involved information on demographics, pre-stroke comorbidities, onset details, imaging features, laboratory results, complications during hospitalizations, interventions, discharge status, and follow-up information. The collation of the database was performed by professional statisticians, and data analysis was carried out after passing the third-party quality control.

The primary outcome of the current analysis was the occurrence of SAP events during hospitalization, and SAP was defined as a pneumonia not incubating during hospital admission and occurring ≥ 48 h after admission in acute stroke populations. Referring to the diagnostic criteria for hospital-acquired pneumonia (HAP), the diagnostic criteria for SAP were as follows (23, 24): the presence of a new or progressive infiltrate in a chest X-ray or CT scan, plus at least two of the following clinical manifestations: (1) fever (T > 38°C) or hypothermia (T < 36°C), (2) leukocytosis [white blood cell (WBC) count > 10 × 10^9^/L] or leukopenia (WBC count < 4 × 10^9^/L), and (3) nursing-recorded purulent airway secretion. Ventilator-acquired pneumonia (VAP), defined as a pneumonia event after ventilation time > 24 h, was excluded from this work.

### Statistical analysis and variable filtration

All statistical analyses were performed using the SPSS software (version 22.0, IBM Corporation, Armonk, NY, USA) and Python (version 3.8.3, Anaconda Distribution, Austin, TX, USA). The current work mainly used the development environment of Jupyter Notebook (version 6.0.3) and invoked the key packaged libraries of NumPy (version 1.18.5), Pandas (version 1.1.5), Scikit-learn (version 0.24.2), SciPy (version 1.5.0), Matplotlib (version 3.4.3), and Lifelines (version 0.26.4). The continuous variables and categorical variables are presented as the mean and standard deviation (SD) or median and interquartile range (IQR) and quantities and percentages.

The screening of variables was performed in the FAHFMU subcohort. As shown in Figure 1B, the study variables were initially screened by univariate analyses. The independent sample Student's *t-*test was used for normally distributed data, the Mann-Whitney U test was used for nonnormally distributed data, and the chi-square test or Fisher's exact test was used for categorical data. All tests in this work were two-sided, and *P* < 0.05 was considered statistically significant. To prevent overfitting, the least absolute shrinkage and selection operator (LASSO) regression was used in multivariate analysis and further performed after univariate analyses. Each continuous variable was standardized before performing LASSO regression to improve generalizability. LASSO regression selects the optimal penalty value *via* the internally installed *k*-fold cross-validation module (*k* = 3) and recursively removes the least important variables by vanishing coefficients. Through the above steps, the independent significant variables had nonzero coefficients in LASSO regression and were selected as candidate variables for ML model derivations.

Survival analysis was additionally performed in this work, in which all-cause death after stroke onset was defined as the observed indicator. The survival time was defined as the time interval from stroke onset to all-cause death or follow-up. The survival curves were plotted using the Kaplan–Meier method, and survival rates were compared using the log-rank test.

### Model derivations and validations

The flow diagram of the model derivations and validations is presented in Figure 1C. The FAHFMU subcohort was randomly split into the training and validation datasets (7:3), which were used for the model derivations and the internal validation, respectively. The model derivations were performed on the candidate variables by six common basic ML algorithms and one additional ensemble model, of which these six well-established algorithms represented various ML frames and are widely accepted at present (19). The ML models were invoked with mature Python packages, including logistic regression (LR), Gaussian naïve Bayes (GNB), random forest (RF), K-nearest neighbor (KNN), support vector machine (SVM), extreme gradient boosting (XGB), and ensemble soft voting model (ESVM). None of these models was uncertain about demonstrating the optimal performance beforehand. In the training process, six basic ML algorithms were independently fitted with the candidate variables and virtual SAP classifications from the training dataset, and model hyperparameters were optimized with the grid-search algorithm to promote model performance. In detail, the grid-search algorithms tune optimal parameters by internally evaluating model performance repeatedly *via* the nested *k*-fold cross-validation module (*k* = 3 in this work). Before the above steps, ML prediction models with different characteristics were generated, and these processes were termed supervised ML. To improve the robustness of ML models, the additional ESVM was derived by incorporating the aforementioned six algorithms. The ESVM is simply a voting system on the weighted classified outputs of the six basic algorithms, and these processes were termed soft voting.

After model derivations, the validation dataset was automatically inputted into the seven models to obtain the predicted classifications in the internal validation. Receiver operating characteristic (ROC) curves were plotted, and the metrics of accuracy, sensitivity, specificity, and area under the curve (AUC) along with 95% CIs were calculated to evaluate the disease discrimination ability of each model. Further supplementary internal evaluation with advanced robustness was performed with *n*-repeated *k*-fold cross-validation (*n* = 3 and *k* = 5 in this work). This method repartitions the FAHFMU subcohort into *k* nonoverlapping folds, where the *k*-1 folds are used for the model derivations and the other fold is used for validation. After *n* repetitions, *n* × *k* combinations are finally generated for robust validation (25).

Furthermore, this work also introduced the external subcohort, which was not involved in variable filtrations and model derivations. In this process, the entire FAHFMU subcohort was considered the training dataset to retrain the prediction models, and the external subcohort was introduced as the exclusive validation dataset. The technical avenue of training and evaluating the models remained the same as above.

## Results

### Participants and characteristics

From January 2015 to April 2021, a total of 909 participants were included in the retrospective cohort of the *Risa-MIS-ICH* study, and 441 of these individuals were excluded due to ventilation > 24 h, ineligible time window, or incomplete data. Finally, 468 individuals (n_FAHFMU_ = 324, n_ACH_ = 144) were included in this work. The overall average age was 60.44 (±12.51) years, and 308 (65.8%) of the individuals were male sex. SAP events during hospitalizations occurred in 135 (28.8%) [n_FAHFMU_ = 97 (29.9%), n_ACH_ = 38 (26.4%)] individuals. The demographic characteristics, clinical manifestations, imaging features, laboratory tests, and prognostic indicators in the FAHFMU and external subcohorts are summarized in Tables 1, 2, respectively. Differences in the analyzed variables between the two centers are shown in Supplementary Table 1.

**Table 1:** Baseline characteristics.

**Variables**	**FAHFMU subcohort**			**External subcohort**		
	**Without SAP**	**With SAP**	***P-*value**	**Without SAP**	**With SAP**	***P*-value**
	**(*n* = 227)**	**(*n* = 97)**		**(*n* = 106)**	**(*n* = 38)**	
Age (years)	58.6 (±11.8)	60.0 (±12.6)	0.370	62.7 (±12.7)	66.0 ± (13.5)	0.182
**Sex**						
Male (*n*)	155 (68.3%)	69 (71.1%)	0.694	59 (55.7%)	25 (65.8%)	0.339
Female (*n*)	72 (31.7%)	28 (28.9%)		47 (44.3%)	13 (34.2%)	
**Pre-stroke history**						
Hypertension (*n*)	163 (71.8%)	74 (76.3%)	0.416	65 (61.3%)	27 (71.7%)	0.329
Diabetes mellitus (*n*)	29 (12.8%)	13 (13.4%)	1.000	4 (3.8%)	3 (7.9%)	0.381
Heart disease (*n*)	8 (3.5%)	4 (4.1%)	1.000	2 (1.9%)	2 (5.3%)	0.284
Smoking (*n*)	59 (26.0%)	24 (24.7%)	0.890	-	-	-
Alcohol abuse (*n*)	59 (26.0%)	23 (23.7%)	0.679	-	-	-
Previous surgery (*n*)	48 (21.1%)	19 (19.6%)	0.768	2 (1.9%)	4 (10.5%)	0.042
**Onset form**						
Neurological dysfunction (*n*)	201 (88.5%)	72 (74.2%)	0.002	91 (85.8%)	31 (81.6%)	0.600
Unconsciousness (*n*)	54 (23.8%)	71 (73.2%)	<0.001	27 (25.5%)	27 (71.1%)	<0.001
Epileptic attack (*n*)	4 (1.8%)	4 (4.1%)	0.246	2 (1.9%)	0	1.000
Headache (*n*)	71 (31.3%)	24 (24.7%)	0.287	91 (85.8%)	21 (55.3%)	<0.001
Others (*n*)	93 (41.0%)	39 (40.2%)	0.903	94 (88.7%)	29 (76.3%)	0.105
Interval time from onset to admission (h)	12.0 (7.0, 24.0)	10.0 (6.5, 16.0)	0.022	3.0 (2.0, 8.3)	3.0 (2.0, 4.5)	0.103
**Admission examination**						
Temperature (°C)	36.5 (36.5, 36.8)	36.7 (36.5, 36.9)	0.115	36.6 (36.5, 36.8)	36.6 (36.5, 36.7)	0.667
Heart rate (min^−1^)	77 (±14)	83 (±17)	0.002	81 (±12)	84 (±14)	0.237
Respiratory rate (min^−1^)	20(19, 20)	20(19, 21)	0.008	20 (20, 20)	20 (20, 20)	0.998
Systolic BP (mmHg)	158 (±24)	162 (±25)	0.145	170 (±24)	174 (±27)	0.473
Dilated BP (mmHg)	93 (±15)	92 (±14)	0.610	100 (±15)	101.8 (±16)	0.453
**Admission GCS Score**						
15 (*n*)	106 (46.7%)	12 (12.4%)	<0.001	80 (75.5%)	10 (26.3%)	<0.001
13–14 (*n*)	77 (33.9%)	33 (34.0%)		8 (7.5%)	5 (13.2%)	
9–12 (*n*)	31 (13.7%)	19 (19.6%)		14 (13.2%)	15 (39.5%)	
5–8 (*n*)	13 (5.7%)	33 (34.0%)		4 (3.8%)	8 (21.1%)	
Hospital costs (thousand CNY)^*^	17.0 (12.5, 25.8)	49.7 (34.4, 91.0)	<0.001	7.7 (6.5, 10.8)	25.1 (14.6, 35.7)	<0.001
Hospital stay (d)^*^	15 (11, 20)	17 (13, 24)	0.003	14 (12, 15)	23 (15, 29)	<0.001
Discharge status^*^						
Home/nursing or rehabilitation (*n*)	96 (42.3%)	46 (47.6%)	0.463	97 (91.5%)	29 (76.3%)	0.022
Care withdrawal or hospital death (*n*)	131 (57.7%)	51 (52.6%)		9 (8.5%)	9 (23.7%)	
Mortality (since onset)^*^						
Survival ≥ 1 year (*n*)	168 (74.0%)	63 (64.9%)	0.009	77 (72.6%)	20 (52.6%)	0.013
3 Months−1 year (*n*)	4 (1.8%)	6 (6.2%)		2 (1.9%)	2 (5.3%)	
<3 Months (*n*)	7 (3.1%)	10 (10.3%)		1 (0.9%)	4 (10.5%)	
Loss of follow-up (*n*)	48 (21.1%)	18 (18.6%)		26 (24.5%)	12 (31.6%)	

* These prognostic variables were not included in further multivariate analysis and model derivations/validations.

SAP, stroke-associated pneumonia; BP, blood pressure; GCS, Glasgow Coma Scale; CNY, Chinese yuan.

**Table 2:** Variables of laboratory results, imaging features, and early clinical interventions.

**Variables**	**FAHFMU subcohort**			**External subcohort**		
	**Without SAP**	**With SAP**	***P*-value**	**Without SAP**	**With SAP**	***P-*value**
	**(*n* = 227)**	**(*n* = 97)**		**(*n* = 106)**	**(*n* = 38)**	
RBC (10^12^ L^−1^)	4.66 (4.30, 4.94)	4.59 (4.15, 4.87)	0.097	4.66 (4.29, 5.08)	4.64 (4.30, 5.11)	0.928
Hemoglobin (g·L^−1^)	142.2 (±14.2)	140.2 (±15.3)	0.278	139.9 (±17.2)	137.9 (±20.2)	0.553
Hematocrit	0.41 (±0.04)	0.41 (±0.04)	0.681	0.42 (±0.05)	0.41 (±0.05)	0.335
WBC (10^9^ L^−1^)^*^	8.52 (6.61, 10.64)	10.17 (7.54, 13.01)	<0.001	8.27 (6.62, 10.83)	9.95 (7.77, 12.28)	0.014
Neutrophil (10^9^ L^−1^)	6.46 (4.42, 8.72)	8.46 (5.49, 11.61)	<0.001	5.86 (4.41, 8.45)	7.49 (5.03, 10.38)	0.016
Lymphocyte (10^9^ L^−1^)	1.29 (0.86, 1.66)	1.04 (0.70, 1.39)	0.001	1.37 (0.99, 1.82)	1.46 (0.99, 1.90)	0.724
Platelet (10^9^ L^−1^)	217.4 (±62.3)	214.8 (±63.3)	0.897	235.1 (±62.4)	221.2 (±55.7)	0.227
PT (s)	11.1 (10.8, 11.7)	11.1 (10.6, 11.9)	0.925	11.3 (10.9, 11.8)	11.4 (10.9, 12.2)	0.307
PT-INR	0.97 (0.94, 1.02)	0.97 (0.93, 1.04)	0.554	0.98 (0.94, 1.03)	0.99 (0.94, 1.07)	0.294
APTT (s)	25.0 (22.2, 27.9)	24.1 (21.8, 27.2)	0.200	25.3 (23.9, 27.1)	24.8 (23.2, 26.8)	0.385
Fibrinogen (g·L^−1^)	2.64 (2.23, 3.04)	2.69 (2.30, 3.13)	0.677	2.62 (2.20, 3.12)	2.68 (2.35, 3.18)	0.607
Serum creatinine (μmol·L^−1^)	67.0 (54.0, 78.3)	66.0 (54.7, 78.2)	0.769	66.0 (57.0, 82.0)	71.5 (58.8, 95.0)	0.098
Serum urea nitrogen (mmol·L^−1^)	5.02 (4.13, 5.94)	5.15 (4.27, 6.59)	0.259	4.85 (4.00, 5.83)	5.10 (4.28, 6.85)	0.276
Serum sodium (mmol·L^−1^)	139.5 (±3.9)	139.9 (±4.6)	0.486	138.7 (±3.5)	138.1 (±3.1)	0.386
Serum potassium (mmol·L^−1^)	3.80 (±0.42)	3.84 (±0.47)	0.474	3.88 (±0.53)	3.92 (±0.61)	0.723
Serum calcium (mmol·L^−1^)	2.28 (±0.54)	2.20 (±0.13)	0.158	2.36 (±0.12)	2.36 (±0.15)	0.802
Serum chloride (mmol·L^−1^)	102.0 (99.0, 105.0)	102.6 (99.0, 105.0)	0.743	100.6 (97.8, 102.5)	99.4 (96.3, 101.4)	0.058
sICH volume (cc)	8.7 (3.9, 17.2)	22.5 (9.4, 37.9)	<0.001	6.8 (3.5, 13.4)	21.7 (6.3, 40.4)	<0.001
Lobar Involvement (*n*)^*^	38 (16.7%)	25 (25.8%)	0.067	23 (21.7%)	12 (31.6%)	0.271
Frontal lobe (*n*)	17 (7.5%)	14 (14.4%)	0.063	8 (7.5%)	5 (13.2%)	0.328
Parietal lobe (*n*)	15 (6.6%)	13 (13.4%)	0.054	10 (9.4%)	4 (10.5%)	1.000
Temporal lobe (*n*)	17 (7.5%)	14 (14.4%)	0.063	10 (9.4%)	9 (23.47%)	0.047
Occipital lobe (*n*)	7 (3.1%)	3 (3.1%)	1.000	5 (4.7%)	2 (5.3%)	1.000
Deep Involvement (*n*)^*^	204 (89.9%)	87 (89.7%)	1.000	87 (82.1%)	31 (81.6%)	1.000
Basal ganglia (*n*)	174 (76.7%)	74 (76.3%)	1.000	66 (62.3%)	29 (76.3%)	0.162
Thalamus (*n*)	56 (24.7%)	33 (34.0%)	0.103	33 (31.1%)	11 (28.9%)	0.841
Corona radiata (*n*)	5 (2.2%)	4 (4.1%)	0.552	6 (5.7%)	6 (15.8%)	0.082
Insular lobe (*n*)	4 (1.8%)	1 (1.0%)	1.000	9 (8.5%)	6 (15.8%)	0.223
Intraventricular involvement (*n*)^*^	60 (26.4%)	47 (48.5%)	<0.001	37 (34.9%)	15 (39.5%)	0.695
Unilateral ventricle (*n*)	26 (11.5%)	13 (13.4%)	<0.001	21 (19.8%)	7 (18.4%)	0.227
Bilateral ventricles (*n*)	33 (14.5%)	33 (34.0%)		15 (14.2%)	8 (21.1%)	
Third ventricle (*n*)	29 (12.8%)	26 (26.8%)	0.003	17 (16.0%)	10 (26.3%)	0.224
Fourth ventricle (*n*)	24 (10.6%)	22 (22.7%)	0.006	14 (13.2%)	7 (18.4%)	0.593
Subarachnoid involvement (*n*)	7 (3.1%)	8 (8.2%)	0.050	3 (2.8%)	1 (2.6%)	1.000
ICU Stay (*n*)	14 (6.2%)	39 (40.2%)	<0.001	0	8 (21.1%)	<0.001
Nasogastric feeding (*n*)	59 (26.0%)	84 (86.6%)	<0.001	11 (10.4%)	24 (63.2%)	<0.001
**Airway support**						
None (*n*)	215 (94.7%)	48 (49.5%)	<0.001	105 (99.1%)	30 (78.9%)	<0.001
Endotracheal Intubation ≤ 24 h or Naso-/oropharyngeal airway (*n*)	2 (0.9%)	13 (13.4%)		0	4 (10.5%)	
Endotracheal intubation > 24 h or tracheotomy (*n*)	10 (4.4%)	36 (37.1%)		1 (0.9%)	4 (10.5%)	
Surgery^*^	18 (7.9%)	50 (51.5%)	<0.001	14 (13.2%)	22 (57.9%)	<0.001
Only sICH evacuation (*n*)	11 (4.8%)	20 (20.6%)	<0.001	0	4 (10.5%)	0.004
Only endoscopic sICH evacuation (*n*)	1 (0.4%)	1 (1.0%)	0.510	0	0	-
Only sICH catheter evacuation (*n*)	0	2 (2.1%)	0.089	9 (8.5%)	7 (18.4%)	0.089
Only EVD approach (*n*)	4 (1.8%)	15 (15.5%)	<0.001	3 (2.8%)	9 (23.7%)	<0.001
Ensemble approaches (*n*)	2 (0.9%)	12 (12.4%)	<0.001	2 (1.9%)	2 (5.3%)	0.573

* These prognostic variables were not included in further multivariate analysis and model derivations/validations.

RBC, red blood cell; WBC, white blood cell; PT, prothrombin time; INR, international normalized ratio; APTT, activated partial thromboplastin time; sICH, supratentorial intracerebral hemorrhage; ICU, intensive care unit; EVD, external ventricular drainage.

### Variable filtration and importance

**Figure 2:**
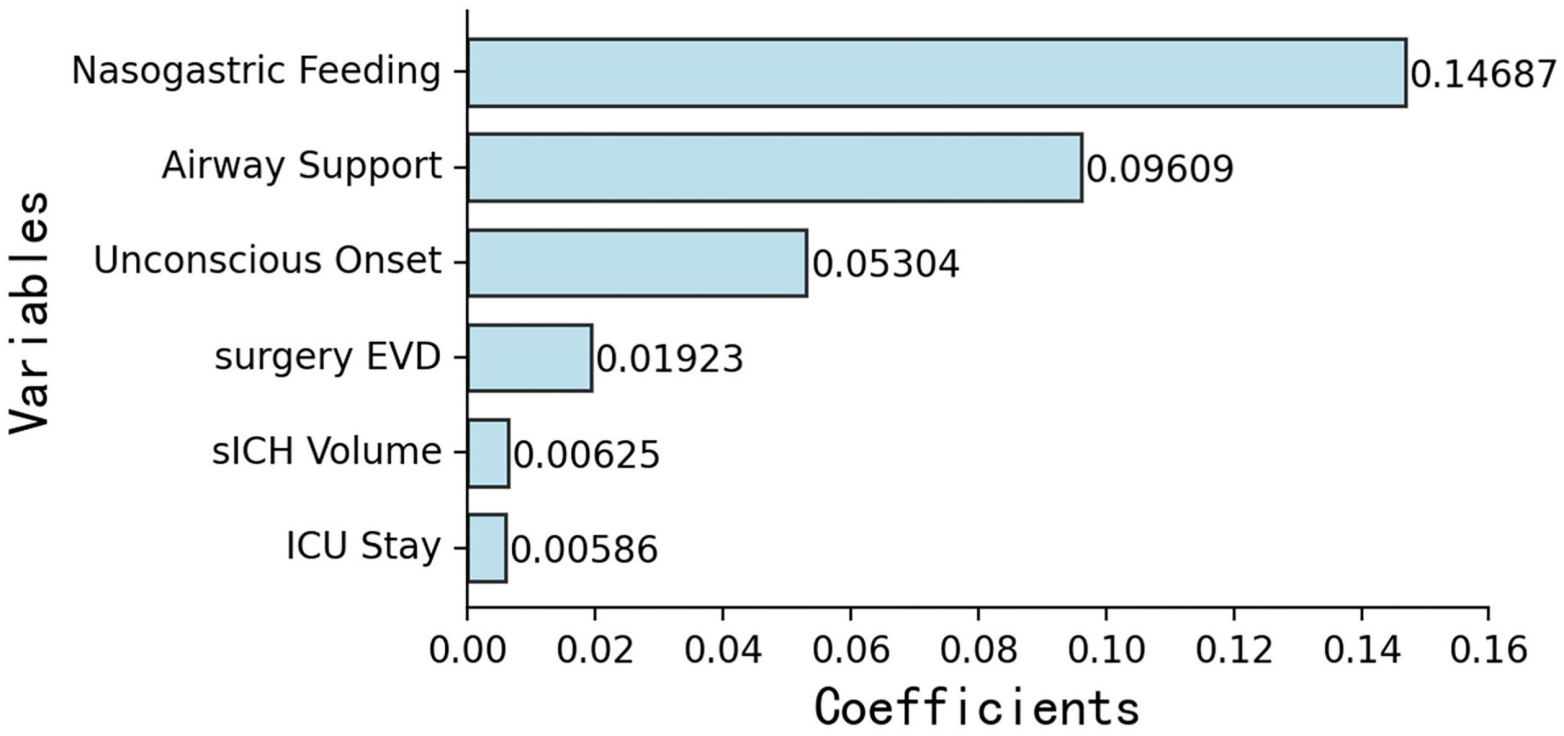
Importance ranking of six independent variables selected by LASSO regression: (1) nasogastric feeding, (2) airway support, (3) unconscious onset, (4) surgery for EVD, (5) larger sICH volume, and (6) ICU stay. EVD, external ventricular drainage; ICU, intensive care unit.

**Figure 3:**
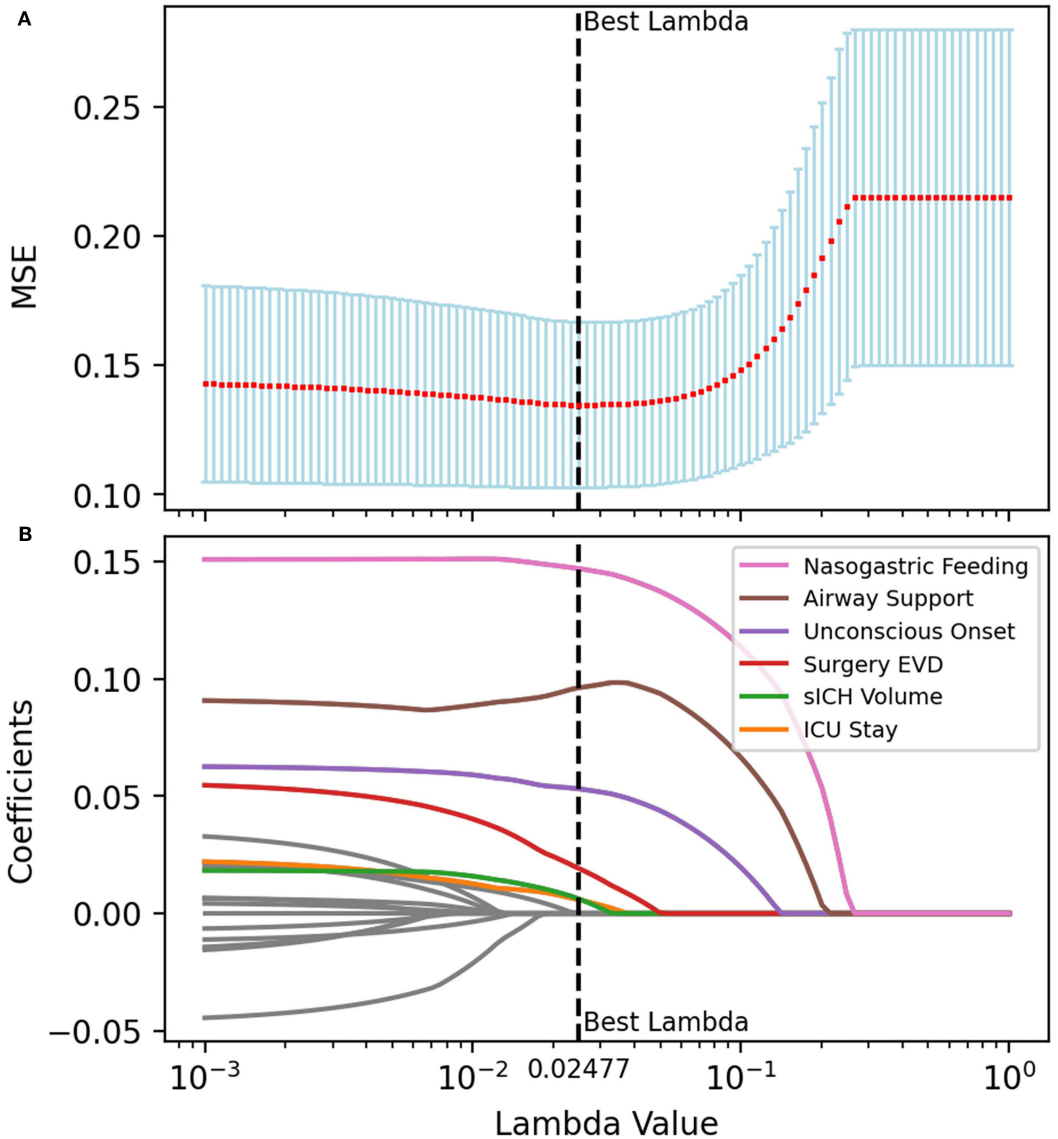
Multivariate analysis and variable filtrations with LASSO regression. The tuning parameter (λ) was selected for the minimized MSE in the LASSO model using 10-fold cross-validation. Features with nonzero coefficients were selected while the previous λ value was applied. **(A)** The MSE was plotted vs. log λ. An optimal λ value of 0.02477 was chosen *via* the minimum criteria and presented as a black vertical dashed line. **(B)** LASSO coefficient profiles of the features. Each colored line represents the coefficient of each feature, and six of them were selected as independent variables when λ equals 0.02477. MSE, mean-square error.

According to previous literature and clinical experience (3–5, 8–16), 70 variables related to the study were retained for subsequent analyses. Twenty-five variables were identified as potential predictive factors for SAP by univariate analysis and further LASSO regression was performed (Tables 1, 2). LASSO regression showed that nasogastric feeding (coefficient = 0.14687), airway support (coefficient = 0.09609), unconscious onset (coefficient = 0.05304), surgery for external ventricular drainage (EVD, coefficient = 0.01923), larger sICH volume (estimated with the ABC/2 formula in imaging, coefficient = 0.00625), and intensive care unit (ICU) stay (coefficient = 0.00586) were considered independent influencing factors of SAP (Figures 2, 3).

### Model performance

**Figure 4:**
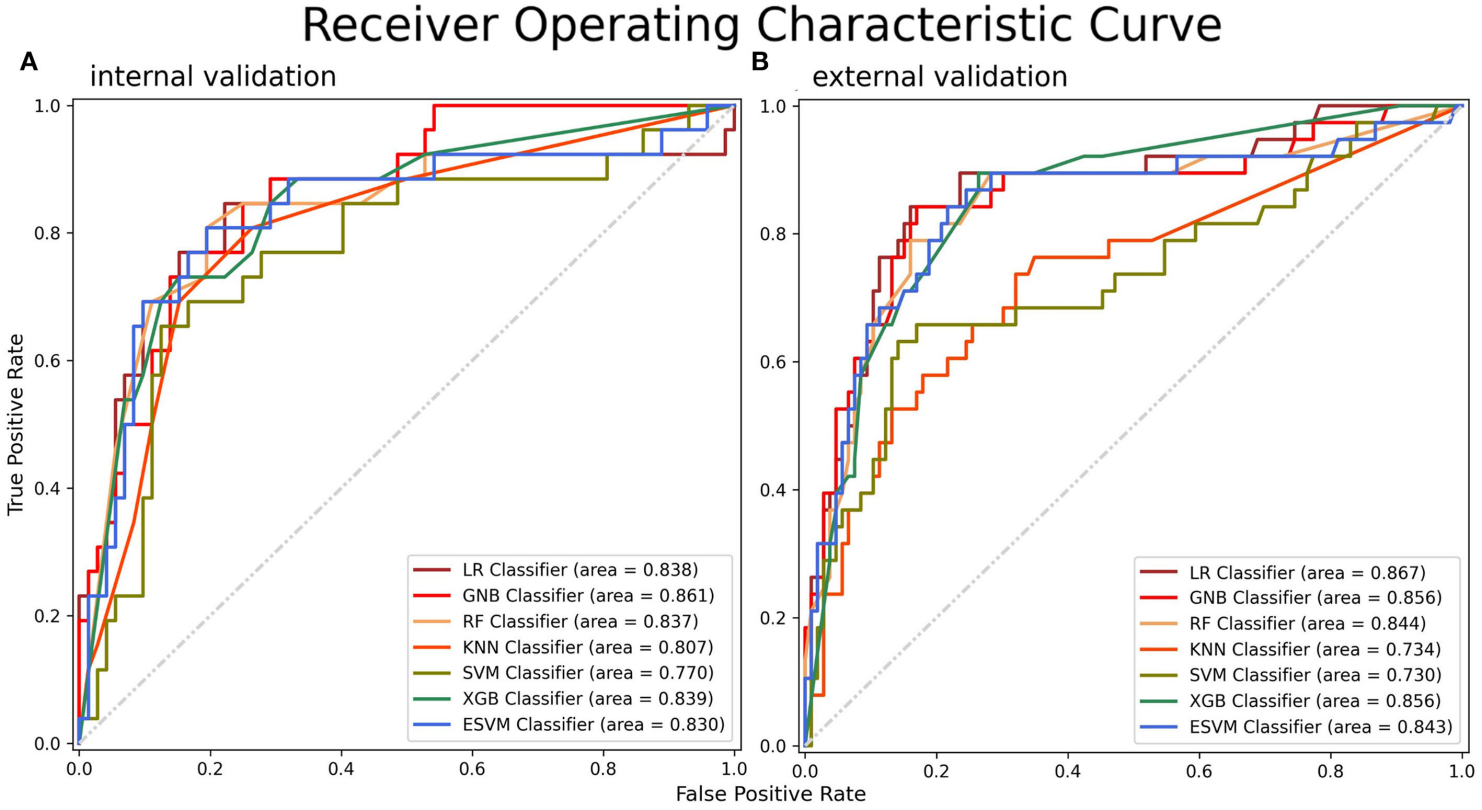
ROC curves for SAP on the **(A)** internal and **(B)** external validation datasets. A greater AUC value indicated a higher predictive ability of the models. ROC, receiver operating characteristic; AUC, area under the curve.

The ROC curves of the seven models built on the internal validation set were shown in Figure 4A. Among the seven models, GNB demonstrated the optimal efficiency to predict SAP with the highest AUC value (0.861, 95% CI: 0.793–0.930), while the ESVM presented the highest accuracy (0.837, 95% CI: 0.764–0.910) and specificity (0.917, 95% CI: 0.862–0.971). The XGB was the most sensitive, with the highest value (0.692, 95% CI: 0.601–0.784) (Table 3A). The decision curve analyses were performed on both training and validation datasets with seven models, as shown in Supplementary Figure 1. The learning curves presented the evolutions of models with different characteristics and are illustrated in Supplementary Figure 2.

**Table 3:** Performance metrics of the ML models in the FAHFMU validation dataset and external subcohort.

	**AUC (95% CI)**	**Accuracy (95% CI)**	**Sensitivity (95% CI)**	**Specificity (95% CI)**
**(A) Internal validation**				
LR	0.838 (0.765, 0.911)	0.827 (0.752, 0.901)	0.615 (0.519, 0.712)	0.903 (0.844, 0.961)
GNB	0.861 (0.793, 0.930)	0.816 (0.740, 0.893)	0.615 (0.519, 0.712)	0.889 (0.827, 0.951)
RF	0.837 (0.763, 0.910)	0.816 (0.740, 0.893)	0.462 (0.363, 0.560)	0.944 (0.899, 0.990)
KNN	0.807 (0.729, 0.885)	0.786 (0.704, 0.867)	0.500 (0.401, 0.599)	0.889 (0.827, 0.951)
SVM	0.770 (0.687, 0.854)	0.786 (0.704, 0.867)	0.500 (0.401, 0.599)	0.889 (0.827, 0.951)
XGB	0.839 (0.766, 0.912)	0.827 (0.752, 0.901)	0.692 (0.601, 0.784)	0.875 (0.810, 0.940)
ESVM	0.830 (0.756, 0.904)	0.837 (0.764, 0.910)	0.615 (0.519, 0.712)	0.917 (0.862, 0.971)
**(B) External validation**				
LR	0.867 (0.812, 0.923)	0.812 (0.749, 0.876)	0.447 (0.366, 0.529)	0.943 (0.906, 0.981)
GNB	0.856 (0.798, 0.913)	0.833 (0.772, 0.894)	0.553 (0.471, 0.634)	0.934 (0.893, 0.975)
RF	0.844 (0.784, 0.903)	0.806 (0.741, 0.870)	0.368 (0.290, 0.447)	0.962 (0.931, 0.993)
KNN	0.734 (0.662, 0.806)	0.778 (0.710, 0.846)	0.395 (0.315, 0.475)	0.915 (0.870, 0.961)
SVM	0.730 (0.658, 0.803)	0.778 (0.710, 0.846)	0.395 (0.315, 0.475)	0.915 (0.870, 0.961)
XGB	0.856 (0.799, 0.913)	0.792 (0.725, 0.858)	0.421 (0.340, 0.502)	0.925 (0.881, 0.968)
ESVM	0.843 (0.784, 0.902)	0.812 (0.749, 0.876)	0.447 (0.366, 0.529)	0.943 (0.906, 0.981)

AUC, area under the curve; LR, logistic regression; GNB, Gaussian naïve Bayes; RF, random forest; KNN, K-nearest neighbor; SVM, support vector machine; XGB, extreme gradient boosting; ESVM, ensemble soft voting model.

Three repeated five-fold cross-validation were established, and a total of 15 combinations were generated from three splits and five-folds. The AUC values of different models from combinations are summarized and presented as heatmaps in Supplementary Figure 3, and all quantified metrics are listed in Supplementary Table 2. In most random states, the ESVM (frequency = 9/15) and XGB (frequency = 8/15) models remained the optimal models in terms of accuracy and sensitivity, respectively. Unlike the results in internal validation, the LR (frequency = 6/15) and RF (frequency = 10/15) models most often had the highest AUC and specificity values, respectively, with robustness.

### External validation

The metrics and ROC curves of each model in external validation are shown in Table 3B and Figure 4B. The LR was superior in AUC value (0.867, 95% CI: 0.812–0.923) in the external validation. While GNB had the highest accuracy (0.833, 95% CI: 0.772–0.894) and sensitivity (0.553, 95% CI: 0.471–0.634), the RF was the most specific (0.962, 95% CI: 0.931–0.993). There was no single algorithm with dominant ability and robustness in the external validation. It is worth mentioning that the ESVM had moderate but robust abilities and achieved AUC, accuracy, sensitivity, and specificity values of 0.843 (95% CI: 0.784–0.902), 0.812 (95% CI: 0.749–0.876), 0.447 (95% CI: 0.366–0.529), and 0.943 (95% CI: 0.906–0.981), respectively, in the external validation. The decision curves for predicting SAP on both FAHFMU and external subcohorts with seven models are illustrated in Supplementary Figure 1.

### Outcome and survival analysis

**Figure 5:**
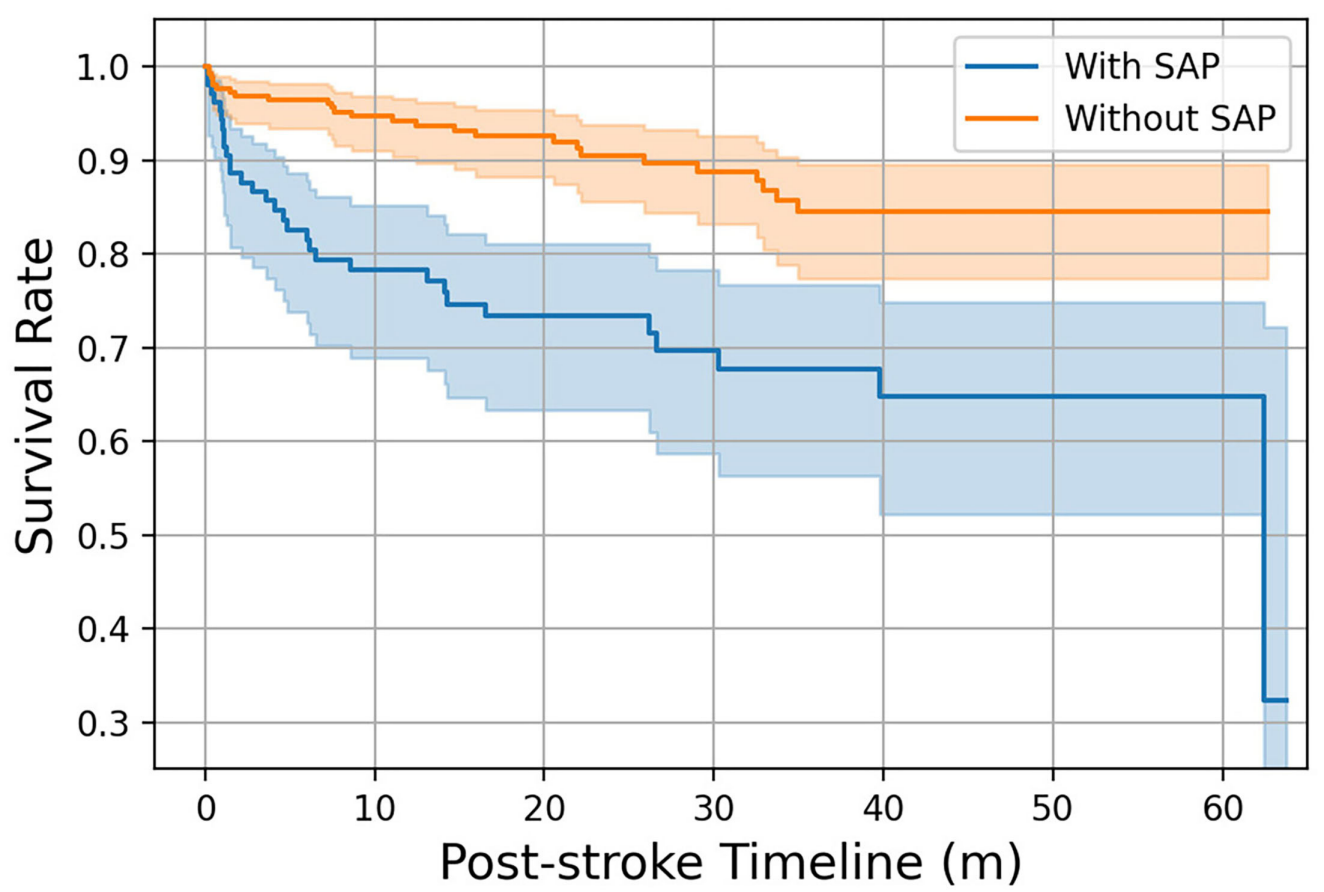
Kaplan–Meier curves of participants with/without SAP over 1-year follow-up. The colored area represents the 95% confidence intervals of the survival rates.

In both the FAHFMU and external subcohorts, participants with SAP suffered from significantly higher hospital costs and prolonged hospital stays (both *P* < 0.001). Three hundred sixty-four (77.8%) of all eligible 468 participants were followed for survival, and 83 (25.3%) of them had experienced SAP during hospitalization. The mean survival times of participants in the two groups were 44.95 ± 2.78 (95% CI: 39.50–50.40) and 55.77 ± 1.26 (95% CI: 53.30–58.25) months, respectively. The median survival times were not available because mortality was < 50%. The 3-month and 1-year survival rates after onset were 86.9 and 78.3% in SAP participants and 96.7 and 94.2% in non-SAP participants. The Kaplan-Meier curves are plotted in Figure 5. There was a significant difference in survival times between the two groups (log-rank χ^2^ = 20.34, *P* < 0.001).

## Discussion

It is critical to identify individuals at high risk for SAP and to further tailor timely prophylactic interventions or therapeutic antibiotics. However, for now, the early prediction of SAP in sICH populations is challenging due to the lack of widely accepted prediction tools, which are important for modern precision medicine and evidence-based medicine (EBM) in this field. Thus, we aimed to derive more effective and automatic sICH-SAP prediction tools in this work. The novel ML prediction models were derived and validated as an attempt to combine artificial intelligence (AI) medical engineering and clinical practice in this field. The major findings were as follows. (1) The incidence rate of sICH-SAP was close to 30%, and the sICH-SAP events significantly contributed to prolonged hospital stays, increased hospital costs, and higher mortality. (2) Six independent predictors for sICH-SAP were identified—nasogastric feeding, airway support, unconscious onset, surgery for EVD, larger sICH volume, and ICU stay. (3) ML prediction models were successfully derived and showed good disease discrimination ability. (4) There was no certain single algorithm with the dominant ability and robustness in cross- and external validations, while the ESVM was considered averaged in metrics and better in robustness in different populations after multiple validations.

Various predictors for SAP were identified in prior literature (4, 5, 8–16). This work screened for independent variables for sICH-SAP events by using univariate and multivariate analyses in the FAHFMU subcohort. Nasogastric feeding, airway support, and unconscious onset were identified as strongly associated risk predictors, which overlapped with the results of previous studies (4, 8–16). Nasogastric feeding and airway support measurement were recognized as SAP predictors, which might bring about secretion disturbances in nasal/oral/tracheal cavities, decreased air filtrations, and even aspiration events (4, 8, 15, 16). These early interventions were secondary to the manifestation of unconsciousness. Previous studies mainly included the ranked variable of the GCS score and rarely adopted the onset manifestations (4, 10, 11, 14–16). In this work, the admission GCS score and unconscious onset were simultaneously introduced into the analyses, and the categorical variable of unconscious onset was independently significant for sICH-SAP. The predictors of larger sICH volume and ICU stay were also reported in previous studies (4, 11, 15) and contributed the least to predicting SAP in this work. The larger sICH volume is a primary factor influencing stroke severity, and ICU stay was a comprehensive intervention secondary to stroke severity and resulted in infectious environments. These aforementioned predictors are usually uncontrollable for actively preventing SAP in clinical practice. However, there were still novel findings in the subgroup analysis that only the surgery for EVD was a significant independent predictor (*P* < 0.001 in FAHFMU/*P* = 0.001 in external subcohorts) of all surgical approaches in this work, while EVD was only previously reported as a univariate factor for overall infections (4). On the other hand, the surgery for sICH catheter evacuation did not significantly contribute to SAP events in any univariate analyses (both *P* = 0.089 in FAHFMU/external subcohorts), which was in accordance with the undifferentiated non-neurologic infections in the *MISTIE* III trial (26). This suggests that we should continuously focus on the stratification of surgical approaches in the prospective cohort of the *Risa-MIS-ICH* study for convincing evidence.

To date, apart from the ICH-APS score, none of the SAP prediction models is widely available in clinical practice (8–13). The validation dataset for the ICH-APS score was obtained from the Chinese National Stroke Registry (CNSR) with an AUC value of 0.74 (95% CI: 0.72–0.75). Both our optimal ML prediction models [internal validation: 0.861 (95% CI: 0.793–0.930); 0.867 (95% CI: 0.812–0.923)] and robust ESVM classifiers [internal validation: 0.830 (95% CI: 0.756–0.904); external validation: 0.843 (95% CI: 0.784–0.902)] achieved higher AUC values, indicating greater predictive ability.

Li et al. (26) developed ML models to predict SAP events in Chinese AIS populations, which presented better performance with the highest AUC value of 0.843 (95% CI: 0.803–0.882) than other AIS-SAP prediction scores (0.835 for A^2^DS^2^, 0.786 for PNA, 0.785 for AIS-APS, and 0.78 for ISAN scores). According to metrics from the literature and this work (27–32), the ML prediction models for SAP showed better performance metrics than traditional scoring systems in both sICH and AIS populations. However, due to incomplete variable collections, horizontal comparisons of different prediction models on the same validation dataset were not possible. Despite the defects, the prediction models usually performed better in internal validation than in external validation due to the intrinsic consistency of original datasets and populational heterogeneity, and the comparisons on their respective original validation datasets usually explained the significance (33).

The published research mainly focused on the mutually separated algorithms. Notably, only the optimal algorithm was mentioned in those articles, although ensemble ML models were reported as successful classifiers with greater performance outcomes in the literature (31, 32). The six basic algorithms used in this work have different characteristics as SAP predictors. RF and LR could identify non-SAP participants better, while XGB could identify SAP participants better. We noted that the predictive ability of one single algorithm was uncertain due to the inconsistent ML algorithmic performance outcomes among the internal/cross-/external validations, and the indeterminacy probably restricted the aforehand model selection and implementation in clinical practice. Therefore, a general and robust model is required for stable predictive ability. Based on the principle of soft voting, we additionally derived ESVM classifier incorporating six basic ML algorithms, which was moderate but surprisingly robust in each metric. Notwithstanding that the occupied machine sources of the ESVM equals the summation of the six basic algorithms, this disadvantage could be ignored by timed training and then *pro re nata* invoking.

Our current work explored the ML for SAP prediction in sICH individuals. During hospitalization, the clinicians could collect the predictive variables and input these variables into an ML model for a predictive suggestion, so that appropriate precautions and interventions would be timely tailored. While the present ML models are semi-automatic and required manual variable input for now. In the coming decades, the internally installed sophisticated algorithms in the EMR system would ceaselessly learn and then calculate the prediction for high-risk individuals in the prospect *via* dynamically evaluating the keyed-in clinical manifestations from clinicians, the resulting values from the laboratory information system (LIS), and the captured data from the picture archiving and communication system (PACS) (29, 34). The ML application may greatly improve the work efficiency of clinicians and the accuracy of judgment results.

We have strengths that deserve comments. An external subcohort and multiple forms of validation were introduced in this work. Therefore, there was populational and algorithmic robustness of convincing results. Based on the aforementioned circumstances, we derived novel ensemble models for generalizability, which showed moderate but robust predictive abilities in different populations and were fit for real-world practice. However, there are limitations that should be acknowledged in this work. First, the observational retrospective design might introduce unmanageable bias. Uncontrollable baseline characteristics in the observational study might confound SAP risks and further model derivations/validations. Second, some important variables were missing due to the retrospective collection of data in this work. The National Institute of Health Stroke Scale (NIHSS) score, uniform CT scan parameters, scanning timing, and other unrecorded details were unreachable in the retrospective cohort of the *Risa-MIS-ICH* study and resulted in the inability to perform horizontal comparisons with external models in this work. Third, not all variables were balanced across the centers, which may bias the results. Although we obtained consistent results based on these imbalanced variables, the influence of the heterogeneity still should not be underestimated. Fourth, there are defects in the deep analyses for SAP. The subgroup analyses on pneumonia severity, radiological features, or pathogenic agents were all absent. A simple overall SAP analysis might be rather rough for complex and heterogenetic pulmonary infections. Future studies on our prospective cohort should continue to resolve the aforementioned problems.

## Conclusions

In this work, the authors derived SAP prediction models with ML algorithms in supratentorial sICH populations from multiple centers and performed multiple validations for effective and robust confirmations. The ensemble model was a novel application in this work and showed robust performance outcomes in different populations. Our attempt indicated that ML application may benefit in the early identification of SAP.

## Data availability statement

The datasets used and/or analyzed during the current work are available from the corresponding author on reasonable request.

## Ethics statement

The studies involving human participants were reviewed and approved by Ethics Committee of First Affiliated Hospital, Fujian Medical University. Written informed consent for participation was not required for this study in accordance with the national legislation and the institutional requirements.

## Author contributions

F-XL, D-ZK, Y-XL, and W-HF designed this study. D-LW and J-CC enrolled participants and supervised in data collecting. YZ, W-HF, and L-YZ performed the machine learning and analyzed the data. Z-YG, R-LC, M-PZ, and Z-FX collected the data for the study. KM performed database management and data cleaning. YZ and Y-XL wrote the initial draft. QH, WH, L-YZ, and F-XL reviewed and edited the paper. D-ZK obtained the funding. All authors read and approved the final manuscript.

## Funding

This work was funded by the Stroke Prevention and Treatment Project of the National Health Commission—Research and Popularization of Appropriate Intervention Technology for the Stroke High Risk Group in China (GN-2018R002), the Fujian Science and Technology Innovation Joint Fund Project (2019Y9118), and the Technology Platform Construction Project of Fujian Province (2020Y2003 and 2021Y2001).

## Conflict of interest

The authors declare that the research was conducted in the absence of any commercial or financial relationships that could be construed as a potential conflict of interest.

## Publisher's note

All claims expressed in this article are solely those of the authors and do not necessarily represent those of their affiliated organizations, or those of the publisher, the editors and the reviewers. Any product that may be evaluated in this article, or claim that may be made by its manufacturer, is not guaranteed or endorsed by the publisher.
