# Supplementary material for: Novel machine learning models to predict pneumonia events in supratentorial intracerebral hemorrhage populations: An analysis of the Risa-MIS-ICH study
Source: Front Neurol. 2022 Aug 25;13:955271. doi: 10.3389/fneur.2022.955271 (PMC9452786; doi:10.3389/fneur.2022.955271)
Supplement: Supplementary file 1 [file Data_Sheet_1.docx]

Supplementary Material

# Supplementary Tables

**Supplementary Table 1**. Comparisons of analyzed variables between centers.

| **Variables** | **FAHFMU Subcohort**  **(n=324)** | **External Subcohort**  **(n=144)** | ***P* value** |
| --- | --- | --- | --- |
| SAP Events | 97 (29.9%) | 38 (26.4%) | 0.442 |
| Age (years) | 59.0 (±12.1) | 63.4 (±12.9) | <0.001 |
| Sex |  |  |  |
| Male (n) | 224 (69.1%) | 84 (58.3%) | 0.027 |
| Female (n) | 100 (30.9%) | 60 (41.7%) |  |
| Pre-stroke History |  |  |  |
| Hypertension (n) | 237 (73.1%) | 92 (63.9%) | 0.049 |
| Diabetes Mellitus (n) | 42 (13.0%) | 7 (4.9%) | 0.008 |
| Heart Disease (n) | 12 (3.7%) | 4 (2.8%) | 0.816 |
| Smoking (n) | 83 (25.6%) | - | - |
| Alcohol Abuse (n) | 82 (25.3%) | - | - |
| Previous Surgery (n) | 67 (20.7%) | 6 (4.2%) | <0.001 |
| Onset Form |  |  |  |
| Neurological Dysfunction (n) | 273 (84.3%) | 122 (84.7%) | 1.000 |
| Unconsciousness (n) | 125 (38.6%) | 54 (37.5%) | 0.838 |
| Epileptic Attack (n) | 8 (2.5%) | 2 (1.4%) | 0.689 |
| Headache (n) | 95 (29.3%) | 122 (77.8%) | <0.001 |
| Others (n) | 132 (40.7%) | 123 (85.4%) | <0.001 |
| Interval Time from Onset to Admission (h) | 12.0 (7.0, 24.0) | 3.0 (2.0, 7.0) | <0.001 |
| Admission Examination |  |  |  |
| Temperature (°C) | 36.6 (36.5, 36.8) | 36.6 (36.5, 36.7) | 0.908 |
| Heart Rate (min^-1^) | 79 (±15) | 82 (±13) | 0.019 |
| Respiratory Rate (min^-1^) | 20 (19, 20) | 20 (20, 20) | <0.001 |
| Systolic BP (mmHg) | 159 (±24) | 171 (±25) | <0.001 |
| Dilated BP (mmHg) | 92 (±14) | 100 (±15) | <0.001 |
| Admission GCS Score |  |  |  |
| 15 (n) | 118 (36.4%) | 90 (62.5%) | <0.001 |
| 13-14 (n) | 110 (34.0%) | 13 (9.0%) |  |
| 9-12 (n) | 50 (15.4%) | 29 (20.1%) |  |
| 5-8 (n) | 46 (14.2%) | 12 (8.3%) |  |
| Hospital Costs (thousand CNY)^*^ | 21.1 (14.0, 43.5) | 9.4 (6.9, 15.4) | <0.001 |
| Hospital Stay (d)^*^ | 15 (11, 20) | 14 (12, 18) | 0.532 |
| Discharge Status^*^ |  |  |  |
| Home/Nursing or Rehabilitation (n) | 142 (43.8%) | 126 (87.5%) | <0.001 |
| Care Withdrawal or Hospital Death (n) | 182 (56.2%) | 18 (12.5%) |  |
| Mortality (since onset)^*^ |  |  |  |
| Survival ≥ 1 year(n) | 231 (71.3%) | 97 (67.4%) | 0.482 |
| 3 Months - 1 year (n) | 10 (3.1%) | 4 (2.8%) |  |
| <3 Months (n) | 17 (5.2%) | 5 (3.5%) |  |
| Loss of Follow-up (n) | 66 (20.4%) | 38 (26.4%) |  |
| RBC (10^12^ L^-1^) | 4.62 (4.26, 4.91) | 4.65 (4.29, 5.09) | 0.377 |
| Hemoglobin (g·L^-1^) | 141.6 (±14.6) | 139.4 (±18.0) | 0.202 |
| Hematocrit | 0.412 (±0.041) | 0.414 (±0.050) | 0.609 |
| WBC (10^9^ L^-1^)^*^ | 8.98 (6.83, 11.19) | 8.92 (6.81, 11.10) | 0.835 |
| Neutrophil (10^9^ L^-1^) | 7.11 (4.68, 9.30) | 6.42 (4.70, 8.85) | 0.372 |
| Lymphocyte (10^9^ L^-1^) | 1.20 (0.84, 1.58) | 1.38 (0.99, 1.84) | 0.001 |
| Platelet (10^9^ L^-1^) | 216.6 (±62.5) | 231.5 (±60.8) | 0.017 |
| PT (s) | 11.1 (10.7, 11.7) | 11.3 (10.9, 11.9) | 0.012 |
| PT-INR | 0.97 (0.94, 1.02) | 0.98 (0.94, 1.04) | 0.098 |
| APTT (s) | 24.6 (22.2, 27.7) | 25.2 (23.7, 27.1) | 0.088 |
| Fibrinogen (g·L^-1^) | 2.65 (2.25, 3.06) | 2.66 (2.26, 3.15) | 0.928 |
| Serum Creatinine (μmol·L^-1^) | 67.0 (54.6, 78.3) | 67.5 (58.0, 82.8) | 0.163 |
| Serum Urea Nitrogen (mmol·L^-1^) | 5.03 (4.15, 6.10) | 4.90 (4.10, 5.98) | 0.476 |
| Serum Sodium (mmol·L^-1^) | 139.6 (±4.1) | 138.5 (±3.4) | 0.003 |
| Serum Potassium (mmol·L^-1^) | 3.81 (±0.44) | 3.89 (±0.55) | 0.135 |
| Serum Calcium (mmol·L^-1^) | 2.25 (±0.46) | 2.36 (±0.13) | 0.006 |
| Serum Chloride (mmol·L^-1^) | 102.0 (99.0, 105.0) | 100.3 (97.3, 102.3) | <0.001 |
| sICH Volume (cc) | 10.9 (4.9, 23.1) | 8.1 (4.1, 20.3) | 0.087 |
| Lobar Involvement (n)^*^ | 63 (19.4%) | 35 (24.3%) | 0.268 |
| Frontal Lobe (n) | 31 (9.6%) | 13 (9.0%) | 0.867 |
| Parietal Lobe (n) | 28 (8.6%) | 14 (9.7%) | 0.727 |
| Temporal Lobe (n) | 31 (9.6%) | 19 (13.2%) | 0.258 |
| Occipital Lobe (n) | 10 (3.1%) | 7 (4.9%) | 0.422 |
| Deep Involvement (n)^*^ | 291 (89.8%) | 118 (81.9%) | 0.023 |
| Basal Ganglia (n) | 248 (76.5%) | 95 (66.0%) | 0.018 |
| Thalamus (n) | 89 (27.5%) | 44 (30.6%) | 0.507 |
| Corona Radiata (n) | 9 (2.8%) | 12 (8.3%) | 0.010 |
| Insular Lobe (n) | 5 (1.5%) | 15 (10.4%) | <0.001 |
| Intraventricular Involvement (n)^*^ | 107 (33.0%) | 52 (36.1%) | 0.527 |
| Unilateral Ventricle (n) | 39 (12.0%) | 28 (19.4%) | 0.080 |
| Bilateral Ventricles (n) | 66 (20.4%) | 23 (16.0%) |  |
| Third Ventricle (n) | 55 (17.0%) | 27 (18.8%) | 0.693 |
| Fourth Ventricle (n) | 46 (14.2%) | 21 (14.6%) | 1.000 |
| Subarachnoid Involvement (n) | 15 (4.6%) | 4 (2.8%) | 0.495 |
| ICU Stay (n) | 53 (16.4%) | 8 (5.6%) | 0.002 |
| Nasogastric Feeding (n) | 143 (44.1%) | 35 (24.3%) | <0.001 |
| Airway Support |  |  |  |
| None (n) | 263 (81.2%) | 135 (93.8%) | 0.001 |
| Endotracheal Intubation ≤ 24 hours or Naso-/Oropharyngeal Airway (n) | 15 (4.6%) | 4 (2.8%) |  |
| Endotracheal Intubation > 24 hours or Tracheotomy (n) | 46 (14.2%) | 5 (3.5%) |  |
| Surgery^*^ | 68 (21.0%) | 36 (25.0%) | 0.399 |
| Only sICH Evacuation (n) | 31 (9.6%) | 4 (2.8%) | 0.017 |
| Only Endoscopic sICH Evacuation (n) | 2 (0.6%) | 0 | 1.000 |
| Only sICH Catheter Evacuation (n) | 2 (0.6%) | 16 (11.1%) | <0.001 |
| Only EVD Approach (n) | 19 (5.9%) | 12 (8.3%) | 0.421 |
| Ensemble Approaches (n) | 14 (4.3%) | 4 (2.8%) | 0.589 |

*These prognostic or general variables were not included in multivariate analysis and model derivations/validations.

**Supplementary Table 2**. Performance of the ML models in cross-validation in the FAHFMU subcohort.

|  | | **AUC (95% CI)** | **Accuracy (95% CI)** | **Sensitivity (95% CI)** | **Specificity (95% CI)** |
| --- | --- | --- | --- | --- | --- |
| Split 1 |  |  |  |  |  |
| LR | Fold 1 | 0.880 (0.801,0.959) | 0.800 (0.703,0.897) | 0.714 (0.604,0.824) | 0.841 (0.752,0.930) |
|  | Fold 2 | 0.916 (0.848,0.983) | 0.908 (0.837,0.978) | 0.875 (0.795,0.955) | 0.918 (0.852,0.985) |
|  | Fold 3 | 0.791 (0.692,0.890) | 0.785 (0.685,0.885) | 0.524 (0.402,0.645) | 0.909 (0.839,0.979) |
|  | Fold 4 | 0.962 (0.916,1.000) | 0.877 (0.797,0.957) | 0.588 (0.469,0.708) | 0.979 (0.944,1.000) |
|  | Fold 5 | 0.834 (0.743,0.925) | 0.781 (0.680,0.883) | 0.500 (0.378,0.623) | 0.929 (0.865,0.992) |
| GNB | Fold 1 | 0.857 (0.771,0.942) | 0.831 (0.740,0.922) | 0.619 (0.501,0.737) | 0.932 (0.871,0.993) |
|  | Fold 2 | 0.909 (0.840,0.979) | 0.892 (0.817,0.968) | 0.750 (0.645,0.855) | 0.939 (0.880,0.997) |
|  | Fold 3 | 0.798 (0.700,0.895) | 0.785 (0.685,0.885) | 0.619 (0.501,0.737) | 0.864 (0.780,0.947) |
|  | Fold 4 | 0.956 (0.906,1.000) | 0.862 (0.778,0.946) | 0.588 (0.469,0.708) | 0.958 (0.910,1.000) |
|  | Fold 5 | 0.830 (0.737,0.922) | 0.766 (0.662,0.869) | 0.455 (0.333,0.577) | 0.929 (0.865,0.992) |
| RF | Fold 1 | 0.878 (0.798,0.957) | 0.815 (0.721,0.910) | 0.714 (0.604,0.824) | 0.864 (0.780,0.947) |
|  | Fold 2 | 0.908 (0.838,0.978) | 0.877 (0.797,0.957) | 0.625 (0.507,0.743) | 0.959 (0.911,1.000) |
|  | Fold 3 | 0.806 (0.710,0.902) | 0.738 (0.632,0.845) | 0.381 (0.263,0.499) | 0.909 (0.839,0.979) |
|  | Fold 4 | 0.947 (0.892,1.000) | 0.846 (0.758,0.934) | 0.529 (0.408,0.651) | 0.958 (0.910,1.000) |
|  | Fold 5 | 0.846 (0.758,0.935) | 0.766 (0.662,0.869) | 0.409 (0.289,0.530) | 0.952 (0.900,1.000) |
| KNN | Fold 1 | 0.846 (0.759,0.934) | 0.831 (0.740,0.922) | 0.571 (0.451,0.692) | 0.955 (0.904,1.000) |
|  | Fold 2 | 0.888 (0.812,0.965) | 0.892 (0.817,0.968) | 0.750 (0.645,0.855) | 0.939 (0.880,0.997) |
|  | Fold 3 | 0.734 (0.626,0.841) | 0.692 (0.580,0.805) | 0.333 (0.219,0.448) | 0.864 (0.780,0.947) |
|  | Fold 4 | 0.888 (0.811,0.965) | 0.815 (0.721,0.910) | 0.412 (0.292,0.531) | 0.958 (0.910,1.000) |
|  | Fold 5 | 0.696 (0.583,0.809) | 0.750 (0.644,0.856) | 0.364 (0.246,0.481) | 0.952 (0.900,1.000) |
| SVM | Fold 1 | 0.758 (0.654,0.862) | 0.754 (0.649,0.859) | 0.667 (0.552,0.781) | 0.795 (0.697,0.894) |
|  | Fold 2 | 0.884 (0.806,0.962) | 0.846 (0.758,0.934) | 0.688 (0.575,0.800) | 0.898 (0.824,0.972) |
|  | Fold 3 | 0.703 (0.592,0.814) | 0.708 (0.597,0.818) | 0.429 (0.308,0.549) | 0.841 (0.752,0.930) |
|  | Fold 4 | 0.784 (0.684,0.884) | 0.815 (0.721,0.910) | 0.412 (0.292,0.531) | 0.958 (0.910,1.000) |
|  | Fold 5 | 0.767 (0.664,0.871) | 0.703 (0.591,0.815) | 0.227 (0.125,0.330) | 0.952 (0.900,1.000) |
| XGB | Fold 1 | 0.825 (0.733,0.918) | 0.769 (0.667,0.872) | 0.714 (0.604,0.824) | 0.795 (0.697,0.894) |
|  | Fold 2 | 0.897 (0.824,0.971) | 0.862 (0.778,0.946) | 0.812 (0.718,0.907) | 0.878 (0.798,0.957) |
|  | Fold 3 | 0.794 (0.695,0.892) | 0.754 (0.649,0.859) | 0.524 (0.402,0.645) | 0.864 (0.780,0.947) |
|  | Fold 4 | 0.939 (0.881,0.997) | 0.846 (0.758,0.934) | 0.765 (0.662,0.868) | 0.875 (0.795,0.955) |
|  | Fold 5 | 0.826 (0.733,0.919) | 0.766 (0.662,0.869) | 0.409 (0.289,0.530) | 0.952 (0.900,1.000) |
| ESVM | Fold 1 | 0.855 (0.769,0.941) | 0.846 (0.758,0.934) | 0.714 (0.604,0.824) | 0.909 (0.839,0.979) |
|  | Fold 2 | 0.916 (0.848,0.983) | 0.908 (0.837,0.978) | 0.812 (0.718,0.907) | 0.939 (0.880,0.997) |
|  | Fold 3 | 0.800 (0.703,0.897) | 0.785 (0.685,0.885) | 0.524 (0.402,0.645) | 0.909 (0.839,0.979) |
|  | Fold 4 | 0.940 (0.882,0.998) | 0.862 (0.778,0.946) | 0.588 (0.469,0.708) | 0.958 (0.910,1.000) |
|  | Fold 5 | 0.821 (0.727,0.915) | 0.781 (0.680,0.883) | 0.500 (0.378,0.623) | 0.929 (0.865,0.992) |
| Split 2 |  |  |  |  |  |
| LR | Fold 1 | 0.825 (0.732,0.917) | 0.769 (0.667,0.872) | 0.706 (0.595,0.817) | 0.792 (0.693,0.890) |
|  | Fold 2 | 0.871 (0.789,0.952) | 0.846 (0.758,0.934) | 0.500 (0.378,0.622) | 1.000 (1.000,1.000) |
|  | Fold 3 | 0.894 (0.820,0.969) | 0.815 (0.721,0.910) | 0.682 (0.569,0.795) | 0.884 (0.806,0.962) |
|  | Fold 4 | 0.848 (0.760,0.935) | 0.800 (0.703,0.897) | 0.556 (0.435,0.676) | 0.894 (0.819,0.969) |
|  | Fold 5 | 0.864 (0.780,0.948) | 0.828 (0.736,0.921) | 0.600 (0.480,0.720) | 0.932 (0.870,0.994) |
| GNB | Fold 1 | 0.855 (0.770,0.941) | 0.769 (0.667,0.872) | 0.588 (0.469,0.708) | 0.833 (0.743,0.924) |
|  | Fold 2 | 0.909 (0.840,0.979) | 0.862 (0.778,0.946) | 0.600 (0.481,0.719) | 0.978 (0.942,1.000) |
|  | Fold 3 | 0.903 (0.831,0.975) | 0.862 (0.778,0.946) | 0.682 (0.569,0.795) | 0.953 (0.902,1.000) |
|  | Fold 4 | 0.839 (0.750,0.929) | 0.815 (0.721,0.910) | 0.556 (0.435,0.676) | 0.915 (0.847,0.983) |
|  | Fold 5 | 0.854 (0.767,0.940) | 0.797 (0.698,0.895) | 0.550 (0.428,0.672) | 0.909 (0.839,0.980) |
| RF | Fold 1 | 0.860 (0.776,0.945) | 0.815 (0.721,0.910) | 0.647 (0.531,0.763) | 0.875 (0.795,0.955) |
|  | Fold 2 | 0.877 (0.797,0.957) | 0.846 (0.758,0.934) | 0.500 (0.378,0.622) | 1.000 (1.000,1.000) |
|  | Fold 3 | 0.882 (0.804,0.961) | 0.846 (0.758,0.934) | 0.591 (0.471,0.710) | 0.977 (0.940,1.000) |
|  | Fold 4 | 0.838 (0.749,0.928) | 0.800 (0.703,0.897) | 0.667 (0.552,0.781) | 0.851 (0.765,0.938) |
|  | Fold 5 | 0.876 (0.795,0.956) | 0.828 (0.736,0.921) | 0.550 (0.428,0.672) | 0.955 (0.904,1.000) |
| KNN | Fold 1 | 0.759 (0.655,0.863) | 0.769 (0.667,0.872) | 0.529 (0.408,0.651) | 0.854 (0.768,0.940) |
|  | Fold 2 | 0.818 (0.724,0.912) | 0.815 (0.721,0.910) | 0.500 (0.378,0.622) | 0.956 (0.905,1.000) |
|  | Fold 3 | 0.799 (0.701,0.896) | 0.708 (0.597,0.818) | 0.455 (0.333,0.576) | 0.837 (0.747,0.927) |
|  | Fold 4 | 0.766 (0.663,0.869) | 0.723 (0.614,0.832) | 0.333 (0.219,0.448) | 0.872 (0.791,0.953) |
|  | Fold 5 | 0.820 (0.726,0.914) | 0.812 (0.717,0.908) | 0.500 (0.378,0.623) | 0.955 (0.904,1.000) |
| SVM | Fold 1 | 0.815 (0.721,0.909) | 0.800 (0.703,0.897) | 0.706 (0.595,0.817) | 0.833 (0.743,0.924) |
|  | Fold 2 | 0.892 (0.816,0.967) | 0.831 (0.740,0.922) | 0.500 (0.378,0.622) | 0.978 (0.942,1.000) |
|  | Fold 3 | 0.830 (0.738,0.921) | 0.846 (0.758,0.934) | 0.727 (0.619,0.836) | 0.907 (0.836,0.978) |
|  | Fold 4 | 0.699 (0.587,0.810) | 0.769 (0.667,0.872) | 0.500 (0.378,0.622) | 0.872 (0.791,0.953) |
|  | Fold 5 | 0.855 (0.768,0.941) | 0.828 (0.736,0.921) | 0.650 (0.533,0.767) | 0.909 (0.839,0.980) |
| XGB | Fold 1 | 0.853 (0.767,0.939) | 0.754 (0.649,0.859) | 0.706 (0.595,0.817) | 0.771 (0.669,0.873) |
|  | Fold 2 | 0.884 (0.806,0.962) | 0.877 (0.797,0.957) | 0.700 (0.589,0.811) | 0.956 (0.905,1.000) |
|  | Fold 3 | 0.871 (0.789,0.952) | 0.769 (0.667,0.872) | 0.636 (0.519,0.753) | 0.837 (0.747,0.927) |
|  | Fold 4 | 0.827 (0.735,0.919) | 0.785 (0.685,0.885) | 0.611 (0.493,0.730) | 0.851 (0.765,0.938) |
|  | Fold 5 | 0.866 (0.783,0.950) | 0.797 (0.698,0.895) | 0.750 (0.644,0.856) | 0.818 (0.724,0.913) |
| ESVM | Fold 1 | 0.841 (0.752,0.930) | 0.815 (0.721,0.910) | 0.647 (0.531,0.763) | 0.875 (0.795,0.955) |
|  | Fold 2 | 0.901 (0.828,0.973) | 0.846 (0.758,0.934) | 0.550 (0.429,0.671) | 0.978 (0.942,1.000) |
|  | Fold 3 | 0.885 (0.807,0.962) | 0.877 (0.797,0.957) | 0.727 (0.619,0.836) | 0.953 (0.902,1.000) |
|  | Fold 4 | 0.836 (0.746,0.926) | 0.800 (0.703,0.897) | 0.500 (0.378,0.622) | 0.915 (0.847,0.983) |
|  | Fold 5 | 0.868 (0.785,0.951) | 0.828 (0.736,0.921) | 0.600 (0.480,0.720) | 0.932 (0.870,0.994) |
| Spit 3 |  |  |  |  |  |
| LR | Fold 1 | 0.839 (0.750,0.929) | 0.785 (0.685,0.885) | 0.611 (0.493,0.730) | 0.851 (0.765,0.938) |
|  | Fold 2 | 0.894 (0.820,0.969) | 0.877 (0.797,0.957) | 0.739 (0.632,0.846) | 0.952 (0.901,1.000) |
|  | Fold 3 | 0.864 (0.781,0.948) | 0.785 (0.685,0.885) | 0.522 (0.400,0.643) | 0.929 (0.866,0.991) |
|  | Fold 4 | 0.887 (0.809,0.964) | 0.846 (0.758,0.934) | 0.571 (0.451,0.692) | 0.922 (0.856,0.987) |
|  | Fold 5 | 0.856 (0.770,0.942) | 0.828 (0.736,0.921) | 0.579 (0.458,0.700) | 0.933 (0.872,0.994) |
| GNB | Fold 1 | 0.855 (0.769,0.940) | 0.846 (0.758,0.934) | 0.611 (0.493,0.730) | 0.936 (0.877,0.996) |
|  | Fold 2 | 0.906 (0.835,0.977) | 0.862 (0.778,0.946) | 0.739 (0.632,0.846) | 0.929 (0.866,0.991) |
|  | Fold 3 | 0.837 (0.748,0.927) | 0.754 (0.649,0.859) | 0.522 (0.400,0.643) | 0.881 (0.802,0.960) |
|  | Fold 4 | 0.898 (0.824,0.971) | 0.846 (0.758,0.934) | 0.571 (0.451,0.692) | 0.922 (0.856,0.987) |
|  | Fold 5 | 0.848 (0.760,0.936) | 0.797 (0.698,0.895) | 0.579 (0.458,0.700) | 0.889 (0.812,0.966) |
| RF | Fold 1 | 0.849 (0.762,0.936) | 0.846 (0.758,0.934) | 0.556 (0.435,0.676) | 0.957 (0.908,1.000) |
|  | Fold 2 | 0.895 (0.820,0.969) | 0.815 (0.721,0.910) | 0.609 (0.490,0.727) | 0.929 (0.866,0.991) |
|  | Fold 3 | 0.858 (0.773,0.943) | 0.754 (0.649,0.859) | 0.435 (0.314,0.555) | 0.929 (0.866,0.991) |
|  | Fold 4 | 0.891 (0.816,0.967) | 0.846 (0.758,0.934) | 0.429 (0.308,0.549) | 0.961 (0.914,1.000) |
|  | Fold 5 | 0.843 (0.754,0.932) | 0.844 (0.755,0.933) | 0.579 (0.458,0.700) | 0.956 (0.905,1.000) |
| KNN | Fold 1 | 0.839 (0.750,0.929) | 0.846 (0.758,0.934) | 0.667 (0.552,0.781) | 0.915 (0.847,0.983) |
|  | Fold 2 | 0.869 (0.787,0.951) | 0.723 (0.614,0.832) | 0.391 (0.273,0.510) | 0.905 (0.833,0.976) |
|  | Fold 3 | 0.763 (0.660,0.866) | 0.800 (0.703,0.897) | 0.522 (0.400,0.643) | 0.952 (0.901,1.000) |
|  | Fold 4 | 0.850 (0.763,0.937) | 0.815 (0.721,0.910) | 0.357 (0.241,0.474) | 0.941 (0.884,0.998) |
|  | Fold 5 | 0.830 (0.738,0.922) | 0.750 (0.644,0.856) | 0.421 (0.300,0.542) | 0.889 (0.812,0.966) |
| SVM | Fold 1 | 0.793 (0.695,0.892) | 0.815 (0.721,0.910) | 0.611 (0.493,0.730) | 0.894 (0.819,0.969) |
|  | Fold 2 | 0.883 (0.805,0.961) | 0.800 (0.703,0.897) | 0.609 (0.490,0.727) | 0.905 (0.833,0.976) |
|  | Fold 3 | 0.803 (0.707,0.900) | 0.800 (0.703,0.897) | 0.522 (0.400,0.643) | 0.952 (0.901,1.000) |
|  | Fold 4 | 0.814 (0.719,0.908) | 0.769 (0.667,0.872) | 0.429 (0.308,0.549) | 0.863 (0.779,0.946) |
|  | Fold 5 | 0.835 (0.744,0.926) | 0.859 (0.774,0.945) | 0.684 (0.570,0.798) | 0.933 (0.872,0.994) |
| XGB | Fold 1 | 0.778 (0.677,0.879) | 0.723 (0.614,0.832) | 0.444 (0.324,0.565) | 0.830 (0.738,0.921) |
|  | Fold 2 | 0.883 (0.804,0.961) | 0.846 (0.758,0.934) | 0.826 (0.734,0.918) | 0.857 (0.772,0.942) |
|  | Fold 3 | 0.833 (0.742,0.924) | 0.800 (0.703,0.897) | 0.609 (0.490,0.727) | 0.905 (0.833,0.976) |
|  | Fold 4 | 0.875 (0.795,0.956) | 0.785 (0.685,0.885) | 0.571 (0.451,0.692) | 0.843 (0.755,0.932) |
|  | Fold 5 | 0.843 (0.753,0.932) | 0.812 (0.717,0.908) | 0.579 (0.458,0.700) | 0.911 (0.841,0.981) |
| ESVM | Fold 1 | 0.864 (0.781,0.947) | 0.831 (0.740,0.922) | 0.611 (0.493,0.730) | 0.915 (0.847,0.983) |
|  | Fold 2 | 0.906 (0.835,0.977) | 0.862 (0.778,0.946) | 0.739 (0.632,0.846) | 0.929 (0.866,0.991) |
|  | Fold 3 | 0.828 (0.736,0.920) | 0.800 (0.703,0.897) | 0.565 (0.445,0.686) | 0.929 (0.866,0.991) |
|  | Fold 4 | 0.889 (0.813,0.966) | 0.846 (0.758,0.934) | 0.571 (0.451,0.692) | 0.922 (0.856,0.987) |
|  | Fold 5 | 0.853 (0.766,0.939) | 0.844 (0.755,0.933) | 0.632 (0.513,0.750) | 0.933 (0.872,0.994) |

# Supplementary Figures


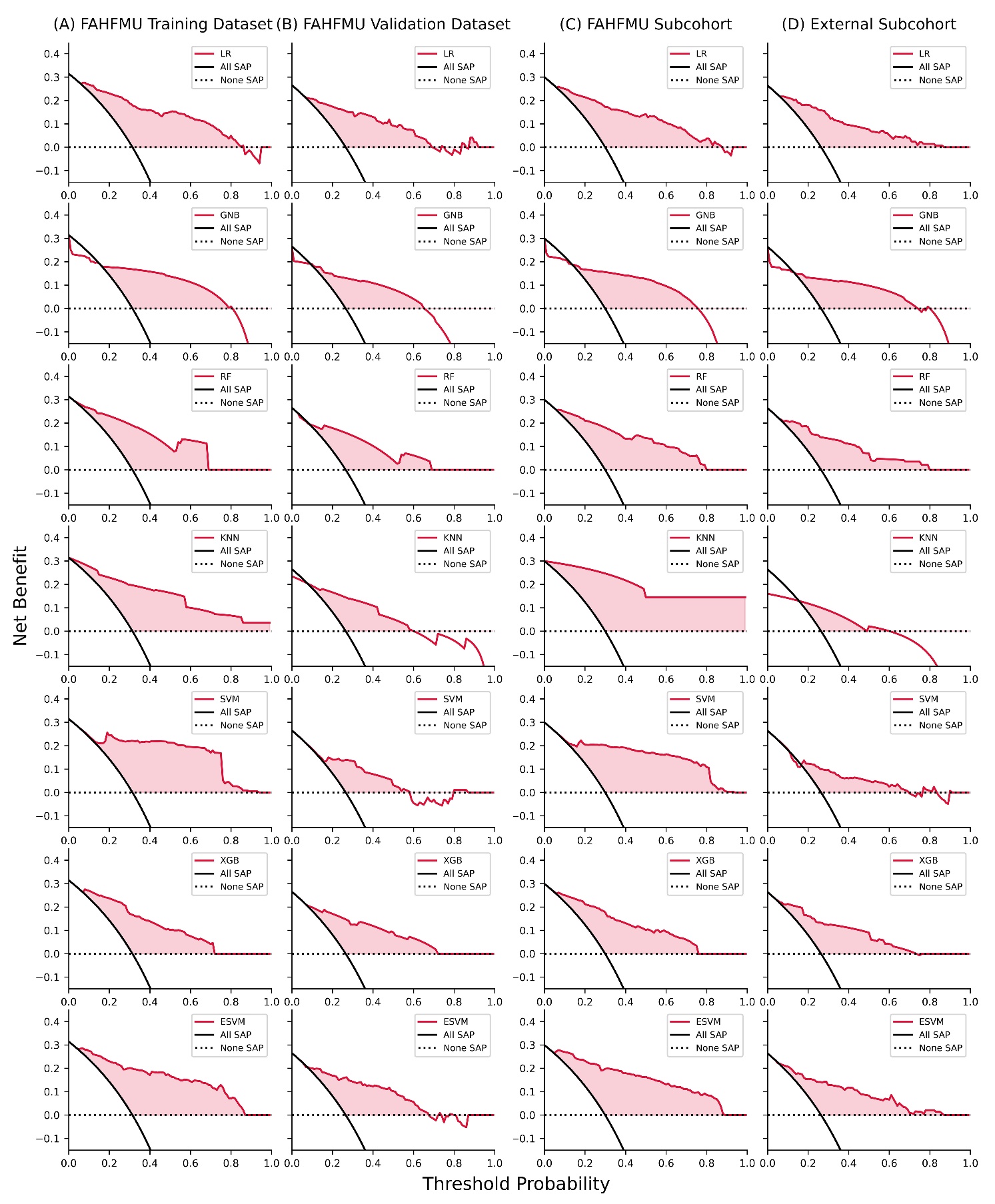


**Supplementary Figure 1.** Decision curve analyses for the SAP prediction on (A) training and (B) validation datasets with internal models and (C) FAHFMU and (D) external subcohorts with external models. The x-axes showed the threshold probability and the y-axes measured the net benefit. The red solid lines represented the different SAP prediction models. The black solid lines represented the assumption that all predictions were possible to undergo SAP. The black dot line represents the assumption that no predictions were associated with SAP.


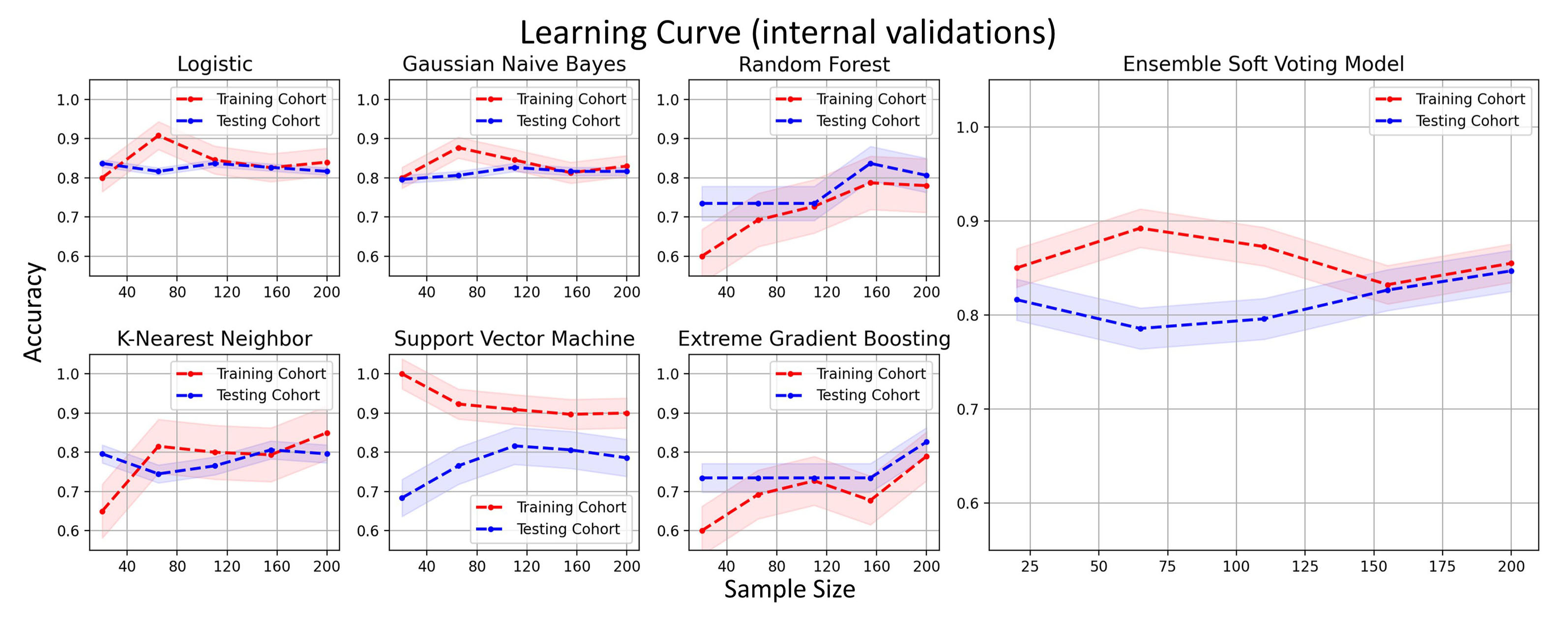


**Supplementary Figure 2.** Learning curves of different ML prediction models in the FAHFMU subcohort. The colored area represents the 95% confidence intervals of the accuracy rates.


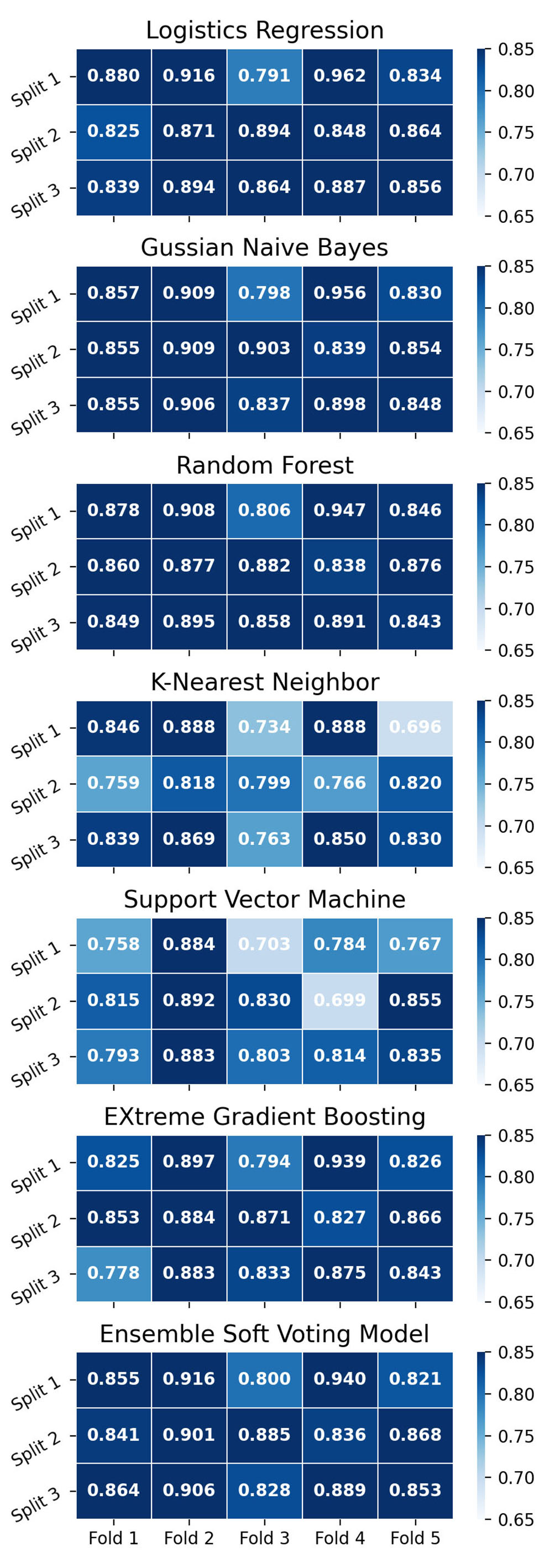


**Supplementary Figure 3.** Heatmaps of different ML prediction models in three repeated five-fold cross-validation. Darker cells represent greater AUC values.
